# Patterns of Bat Distribution and Foraging Activity in a Highly Urbanized Temperate Environment

**DOI:** 10.1371/journal.pone.0168927

**Published:** 2016-12-28

**Authors:** Jennifer J. Krauel, Gretchen LeBuhn

**Affiliations:** Department of Biology, San Francisco State University, San Francisco, California, United States of America; University of Fribourg, SWITZERLAND

## Abstract

Understanding how to manage biodiversity in urban areas will become increasingly important as density of humans residing in urban centers increases and urban areas expand. While considerable research has documented the shifts in biodiversity along urbanization gradients, much less work has focused on how characteristics of dense urban centers, effectively novel environments, influence behavior and biodiversity. Urban bats in San Francisco provide an opportunity to document changes in behavior and biodiversity to very high-density development. We studied (1) the distribution and abundance of bat foraging activity in natural areas; and (2) characteristics of natural areas that influence the observed patterns of distribution and foraging activity. We conducted acoustic surveys of twenty-two parks during 2008–2009. We confirmed the presence of four species of bats (*Tadarida brasiliensis*, *Myotis yumanensis*, *Lasiurus blossevillii*, and *M*. *lucifugus*). *T*. *brasiliensis* were found in all parks, while *M*. *yumanensis* occurred in 36% of parks. Results indicate that proximity to water, park size, and amount of forest edge best explained overall foraging activity. Proximity to water best explained species richness. *M*. *yumanensis activity* was best explained by reduced proportion of native vegetation as well as proximity to water. Activity was year round but diminished in December. We show that although bats are present even in very densely populated urban centers, there is a large reduction in species richness compared to that of outlying areas, and that most habitat factors explaining their community composition and activity patterns are similar to those documented in less urbanized environments.

## Introduction

Well-documented trends of increasing urbanization represent a major impact on most ecological systems: humans move to urban areas, as human density increases, urban biodiversity decreases and becomes more homogenous, and ecosystem services may decline [1]. Most research has focused on describing characteristics of urbanization gradients, documenting a common pattern of decreasing species richness and abundance with increasing urbanization [2, 3]. Meanwhile, as suburban corridors fill in, core urban areas are becoming less green and more densely populated [4], with unknown effects on related ecosystem services [5, 6]. Thus, if we wish to maintain biodiversity there is an urgent need to understand processes and mechanics involved in maintaining biodiversity in densely populated urban areas (2).

Core urban areas, characterized by high-density housing or industrial areas interspersed with green spaces of varying degrees of naturalness, represent an opportunity to preview ecological processes expected to become common in many future cities. These areas are novel ecosystems in several ways: they are saturated with light, so patterns of nocturnal biodiversity are likely to be significantly disrupted [7, 8]; the food supply is quite different from natural areas, with an abundance of resources even in winter for granivores or other generalist animals [9, 10] but a reduced supply of invertebrates for insectivores [11–13]; an abundance of potential roosts [14, 15]; a harsh matrix between foraging areas [16, 17]; and increased warmth in all seasons [18].

Bats offer an interesting opportunity to study high-density, urban ecology for several reasons. First, because they are volant, bats may be more tolerant of a harsh matrix than other mammalian species. Second, unlike birds, bat communities normally do not include introduced species, so they are less likely to be affected by homogenization [19]. Finally, they are not influenced by regular direct human interactions that might affect community composition such as supplemental feeding or interruption of foraging behavior [20, 21]. Thus, studies of bat communities in densely populated urban areas may offer insights more directly related to the urbanized aspects of the environment. Studies have demonstrated dominance of a few species in core urban areas [22–26], often species adapted to foraging in open areas or above clutter [27], which would enable bats to find suitable foraging areas despite considerable light, noise, and distance between foraging locations or between roost and foraging sites. Jung and Threlfall [28] have shown that a high degree of urbanization leads to declines in intensity of habitat use by bats. Thus, the interesting question is what leads to that decline.

In most habitats, bats are thought to be limited more by roost availability than food availability [29], but this may not be the case in urban areas [30]. Urban structures may provide a greater variety of roosting options than are found under natural conditions [26, 31, 32]. Bat activity is often directly related to insect activity and mass [22, 33], and reduced insect diversity and abundance relative to surrounding areas [34, 35] suggests that urban bats may be limited by access to food resources and thus ecological factors influencing insect abundance drive bat distribution in these areas [26]. Studies of bats that forage in urban areas have reported a correlation between activity and amount of forest edge [36, 37] and proximity to water [24, 38–40]. Isolation of the response of different taxa to specific habitat factors in core urban habitats may provide insights into responses of individual species or communities to expected future intensification of urban pressure.

San Francisco offers an excellent opportunity to study bat activity in these dense, core urban habitats. A relatively small land area (4687 ha), it is the second-most densely populated area in North America, after New York City [41]. With salt water on three sides, the San Francisco peninsula can be an effective barrier for even volant species, although some bat species have been known to cross open water during migration [42, 43]. San Francisco also has no significant amount of agricultural area surrounding it; the approach over the peninsula passes through suburban areas and through natural and access-limited land owned by the San Francisco water district (9307.77 ha) and the Mt. San Bruno natural area (941.30 ha). Previous studies in the city and in surrounding counties, as well as museum specimens, show the regional species pool containing up to 15 species of bats, many of which might reasonably be found inside the city (Table 1). Most other urban areas studied have not controlled for the influence of surrounding suburban or agricultural areas in the landscape matrix [22, 36]. Thus, the physical isolation from surrounding areas and the density of development make San Francisco a unique opportunity to isolate the mechanisms affecting the bat community in the urban core area.

**Table 1:** Regional bat species pool for San Francisco and surrounding counties.

Bat Species	Alameda	Contra Costa	Marin	San Francisco	San Mateo
**Vespertilionidae**					
*Antrozous pallidus*	A, B	A, B	A, B		A, B, E
*Eptesicus fuscus*	A, B	B	A, B, C, D	C, D, F	A, B, E, G
*Lasionycteris noctivagans*	A, B	B	A, B, D	D	G
*Lasiurus blossevillii*	A, B	A, B	A, B, D	A, B, D, F, H	A, B, C, E, G
*Lasiurus cinereus*	A, B	A, B	A, B, D	A, C, D, F	A, B, E, G
*Myotis californicus*	A, B	B	A, B, C, D*	B, D*, F*	B, E, G*
*Myotis evotis*	B	B			A, B, E
*Myotis lucifugus*			D	D, H	E
*Myotis thysanodes*	B		B, D	D	A, B, E, G
*Myotis volans*			A		A, B, E
*Myotis yumanensis*	A, B	B	B, D*	A, B, D*, F*, H	A, B, E, G*
*Parastrellus hesperus*					
*Corynorhinus townsendii*	A		A, B		A, E
**Molossidae**					
*Eumops perotis*			A		
*Nyctinomops macrotis*		B			B
*Tadarida brasiliensis*	A, B	A, B	A, B, D	A, B, C, D, F, H	A, B, E, G

Species records for San Francisco and surrounding counties. In December 2009, we searched the electronic databases of the following natural history collections for additional voucher records from San Francisco and surrounding counties: (A) California Academy of Sciences, San Francisco, California, (B) the Museum of Vertebrate Zoology, Berkeley, California, and (C) the National Museum of Natural History, Smithsonian Institution, Washington, DC. Species presence is also noted from the following studies: (D) [44], (E) [45], (F) [46], (G) [47], and (H) Present study.

* Study did not distinguish acoustically between *M*. *californicus* and *M*. *yumanensis*.

In this paper, we relate foraging area characteristics to the diversity of bat communities and provide baseline data on bat species diversity and community composition in a highly dense urban area. We predict that if San Francisco bats are food-limited rather than roost-limited, like other urban bat communities, we will find (1) Reduced species richness in San Francisco than in surrounding areas, with species present representing a subset of the regional species pool, (2) Dominance of one or two species, probably bats adapted for foraging above clutter and over larger areas, and (3) High importance of habitat factors related to insect availability.

## Methods

### Study area

San Francisco’s climate is defined as coastal Mediterranean with dry mild summers and wet mild winters [48]. This particular climate has a dry season lasting typically from May until October and a wet season from November until April. San Francisco receives an average of 95% of its annual rainfall from late October through March [49]. Wind and fog are common year-round and may influence bat activity as bats have been shown to be less active in moderate to strong winds [50, 51] and fog [52, 53].

**Fig 1:**
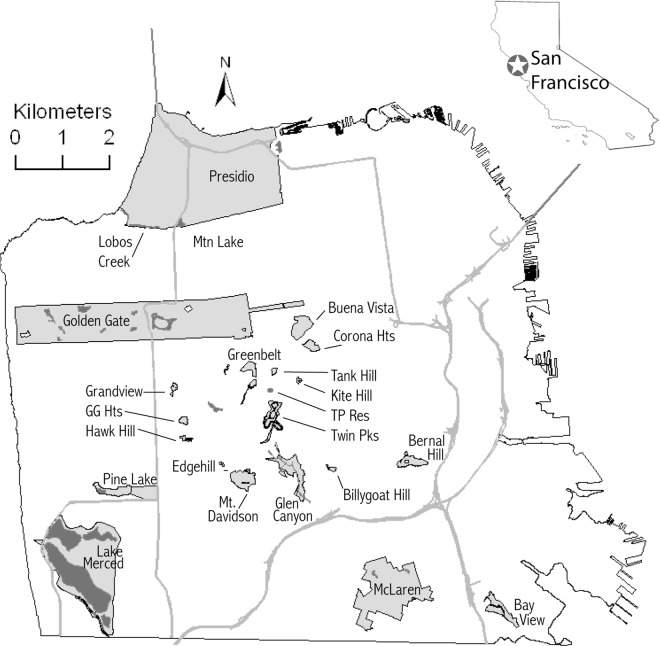
Map of San Francisco, California parks surveyed in 2008–2009.

The San Francisco landscape consists of patches of very highly developed land, urban residential neighborhoods with varying levels of vegetation, and mostly isolated patches of parkland. Parks with natural areas in the city consist of a set of federally managed areas, collectively called the Golden Gate National Recreation Area (GGNRA), and a set of 31 parks managed by the city that have areas designated as Significant Natural Areas (hereafter called “natural areas”) ranging in size from 0.12 ha to over 120 ha. San Francisco residents and visitors have access to these natural areas for recreational purposes such as hiking, nature watching, and dog walking. Natural areas are defined as having remnant fragments of the Franciscan landscape [54] that are largely unchanged by human activity. However, these undeveloped natural areas are not pristine and many are dominated by non-native plant species. They also contain a mosaic of coastal scrub, perennial grasses, chaparral, riparian wetlands, and native patches of coastal live oak and laurel trees, which support many sensitive plant and animal species and are an important refuge for urban biodiversity [55]. While bats may be able to forage in all portions of the city, we expected that we would measure activity and richness most efficiently and effectively by sampling in natural areas. Surveys done in addition to the present study, for example in residential areas, indicate that the only species regularly detected in more urbanized areas within the city was *T*. *brasiliensis*. We studied 22 parks (Fig 1, S1 Table), of which 15 were chosen to enable comparison with 2 earlier studies on biodiversity conservation in natural areas [56, 57]. Seven additional parks were added to provide a suitably large sample size for regression-based modeling. Sites were at least 400m apart.

### Bat activity and diversity

We sampled bat activity at each site using stationary acoustic detectors that recorded from dusk until dawn. Nineteen of the 22 park sites were surveyed four times, one night in each of four quarterly periods: April 26-May 30, July 21-August 4, December 2–9 2008, and March 13–20 2009. Three additional parks were added after the first quarterly period and sampled during each period thereafter. These four periods were chosen to identify resident summer and winter bats as well as possible spring and fall migrants. Recording dates were as close together as possible during each survey period, often on subsequent nights, at up to four sites per night. We tried to minimize variation in weather conditions among nights within survey periods, and only sampled on nights with winds less than 20 mph. Parks were visited in random order each period except for those in the southern end of the city, which for logistical purposes were always surveyed together. To determine the sampling site within each park, we used GIS software (ArcMap v9.2, Esri, Redlands, California) to identify tree line and water edges which would be most likely to attract foraging bats within each park [36, 58, 59]. Multiple random locations were generated along those edges in each park [60] and were sequentially evaluated on site. We selected the first location for each park where a detector could be mounted in a secure manner. Detectors were set at heights ranging from 1–3 m, depending on the location, and facing perpendicular to the expected bat foraging area or corridor. While detectors at water edges may cover a wider area than those at tree lines, we attempted to minimize this effect by concealing water-edge-based detectors in vegetation similar to that available at tree line sites. Because weather data was not available for individual sites, weather variables were obtained at a single citywide level for each night [61].

We collected acoustic samples using Pettersson D240X ultrasonic acoustic detectors (Pettersson Elecktronik AB, Upssala, Sweden) connected to iRiver IPF digital recorders (iRiver America, Vancouver, Washington). Detectors were assigned randomly to each park night and were deployed inside a waterproof casing at each site before dusk and retrieved after dawn. We configured detectors to automatically trigger upon detection of ultrasonic noise (high level, HF source) and to record time-expanded 1.7s echolocation call sequences in separate files on the recorder, at 44.1 kHz and 160 kbs, We calibrated detectors using an ultrasonic emitter (Bat Chirp, T. Messina, Las Vegas, Nevada) at the start of the study and periodically thereafter. We visually analyzed the recorded bat call files using SonoBat software (SonoBat v2.6. SonoBat, Arcata, CA., default settings) to verify presence of one or more bat calls in a sequence [62, 63]. Files without recognizable bat calls (e.g. only insect activity or wind noise) were discarded, and the number of remaining files with recognizable call sequences was used as the measure of total bat activity at a site [63]. This number does not represent the number of individuals in the area, but rather a relative measure of bat activity at a particular location [36, 64]. While it is sometimes possible to infer the presence of multiple bats within a single call file using inter-call intervals, it is not possible to accurately count the number of individual bats in each call file. Thus the number of call files should be interpreted as a measure of relative activity duration rather than quantity of concurrent activity.

For species identification, we used Sonobat to visually inspect one or more calls in a sequence and compare them to call libraries of known species from the western United States provided with the SonoBat software [65, 66]. We used only call sequences with multiple high quality search phase bat calls [67]. We selected the highest quality call from each sequence and identified calls to species based on low frequency, high frequency, characteristic frequency (the frequency of the call at its lowest slope, or the lowest frequency for consistent FM sweeps), frequency with the greatest power, call duration, and upper and lower call slope [68, 69]. For some species, such as *Lasiurus blossevillii*, variation in call attributes between calls in a sequence is an important characteristic and in those cases the entire call sequence was considered [69]. In addition, we compared calls to those of known species from the western United States using call libraries including those provided with the SonoBat software. Dr. Joseph Szewczak, Humboldt State University, California, independently reviewed a subset of the calls to validate the procedure.

There can be considerable overlap between call parameters in species using lower-frequency calls [67]. Typical *Tadarida brasiliensis* foraging calls were the most common in the study area, but *Eptesicus fuscus* and *L*. *cinereus* are acoustically similar and have been reported in San Francisco [46] as well as in the surrounding areas (Table 1). While no calls recorded from this study were a strong match for typical *E*. *fuscus* calls, a very small number were highly suggestive of *L*. *cinereus*. However, because attributes of some of their calls can overlap strongly with those of *T*. *brasiliensis*, we conservatively assigned all to *T*. *brasiliensis*, the dominant and most acoustically variable species of that group.

### Park characteristics and analysis

We identified potential habitat-related explanatory variables for each park site based on those reported in other studies of urban bats, including park size (“Pk Size”) [22], amount of forest edge (“Edge”) [8, 36, 37], percent native habitat (“Native”) [70], distance to the nearest large park (“Lg Pk”) [22], and distance to nearest water (“Water”) [39, 40, 71]. Models including distance-based variables are more accurate than those including only within-park variables [72]. We measured park size, proximity to water, and proximity to the nearest large park (> 100 ha) using ArcMap. Proximity was measured as the distance from the recording location to the edge of the nearest body of water or large park. We determined the area of native vegetation and the amount of forest edge for each park using GIS-based data supplied by the San Francisco city parks Natural Areas program [73]. While most tree-covered areas in San Francisco are smaller than may be generally considered as “forest”, we defined forest edge in this study to be the perimeter distance around polygons outlining tree-covered areas inside a park [8]. Most parks were clearly delineated by highly developed residential or commercial areas, but five parks were adjacent to golf courses, heavily wooded neighborhoods, or other large open space that could represent contiguous foraging areas to bats. For example, Fort Funston Park, the San Francisco Zoo, and the Harding Park and the Olympic Club golf courses are immediately adjacent to the Lake Merced site. We used ArcMap to re-calculate park size to include those open spaces to better reflect available foraging area within the site, and revised the estimate for forest edge to include polygons outlining trees in these open spaces. Golf courses in San Francisco are not permitted to use pesticides, so we considered them as potential sources of insects for foraging bats. All golf courses adjacent to study parks were similarly forested, so we estimated the amount of forest edge in golf courses for which we had no detailed GIS layers by computing a ratio of edge to area for a representative golf course for which we had forest edge metrics available (Presidio). No additional open water was included in the adjacent open spaces or golf courses. Additional site details are available in S1 Table.

Basic correlations between activity and explanatory variables were examined in JMP (JMP, 2009. SAS Institute, Cary, NC). To model which of these park characteristics are best at explaining differences in bat activity between parks, we built *a priori* models using all five explanatory variables (park size, amount of forest edge, distance to nearest large park, distance to water, and percent native vegetation) based on linear regression using R [74]. To measure bat activity, we used the median number of files containing recognizable bat calls across all recording nights from each park divided by the number of sampling periods for that park.

We modeled total foraging activity as well as species-specific activity for the two most common bats, *T*. *brasiliensis* and *M*. *yumanensis*. Foraging activity data were best fit by negative binomial models [75]. One park site, Lobos Creek in the Presidio, was removed from *M*. *yumanensis* regression models as an outlier due to extremely high activity levels on one night. A second park site, Greenbelt, was removed from total and *T*. *brasiliensis* activity models as an outlier because the site was dominated by eucalyptus forest creating a very different interior site configuration. This site also had predominately windy and foggy weather conditions across all seasons. These issues resulted in an abnormally low number of high-quality calls. We transformed explanatory variables to approach normality and screened them for multicollinearity using Pearson correlation matrices and the variance inflation factor [76]. We examined the pattern of the residuals for each regression model and found no evidence to suggest that generalized linear regression was not the appropriate model for these data. We analyzed median total activity overall and of *T*. *brasiliensis* and *M*. *yumanensis* separately using the generalized linear regression function negbin in package aod [77] including all 5 explanatory variables. In generalized linear regression it is necessary to estimate the amount of variation in the data explained by a model using D^2^, a deviance-based analogue of R^2^ [78]. We estimated D^2^ using the package modEvA [79]. We used model analysis and multimodel inference to determine the models that best described foraging activity and species richness [76]. Because of the relatively small number of parks, no more than three variables were included in each model. We used the package MuMIn [80] to compute 2nd-order Akaike’s Information Criterion (QAICc), calculate Akaike weights, select the most parsimonious models given the data, and compute model-averaged estimates for parameters [76]. QAICc is a version of AICc for overdispersed count data sets. Models with lower QAICc values are most parsimonious and represent a better fit with observed data, and QAICc model weight is an indicator of relative likelihood that a particular model explains the observed activity.

We calculated the extent of sampling coverage using EstimateS [81]. To measure species richness, we evaluated the number of separately identified species per park night. The total richness value for each park represents the cumulative number of species recorded in that park over the course of the year. Since species richness could not be transformed to approach a distribution enabling a generalized linear regression analysis, we modeled species richness predictors with a cumulative link model ordered multinomial regression using the clm function from the Ordinal package [82] and evaluated models as for activity. Exploratory analyses of effects of temperature and other climate variables showed no significant effect on activity or species richness within survey periods and were not pursued further. We considered seasonal effects on activity across parks between survey periods by using repeated measures ANOVA and Tukey post-hoc tests with survey periods as the explanatory factor, using the glm.nb function followed by the glht function in library multcomp [83]. Because activity varied considerably with park size, we controlled for effect of park size on seasonal variation by including a size group categorical variable, where large parks > 1,000 ha, medium parks > 100 ha, and small parks < 100 ha.

Access to study sites for acoustic sampling was granted by permits issued by the U.S. National Park Service (Presidio sites), the San Francisco Fire Department (Twin Peaks Reservoir), and the San Francisco Natural Areas Program (all other sites). This study was conducted in accord with San Francisco State University IACUC protocol #A7¬003.

## Results

From May 2008 through April 2009, on 85 park nights over at least 700 recording hours, we recorded 5,585 bat passes representing at least 4 separate bat species. We classified 4,700 bat passes (84%) as those of *T*. *brasiliensis*, and 831 passes (14.9%) of *Myotis yumanensis*. We also confirmed recordings of *Lasiurus blossevillii* (16 passes) and *M*. *lucifugus* (6 passes). Sampling coverage was complete over 85 samples, with the Chao1 and Chao2 estimators of mean 4 species, 95% CL upper limit = 4.48. Species richness estimators do not predict more species with additional sampling effort for this dataset.

Models containing proximity to water, amount of forest edge or park size best explained overall foraging activity, explaining 61, 55, and 49% of activity respectively (Tables 2 and 3), although multimodel inference across all models of activity shows that no single variable was significant. Because *T*. *brasiliensis* represented 84% of all bat activity, the species-specific model (Table 2) was similar but showed a slightly stronger effect. Amount of forest edge was the most important variable, followed by park size and then proximity to water (Table 3). Activity of *M*. *yumanensis* was best explained by lower proportions of native vegetation and proximity to water, contributing 37 and 29% of model variation (Table 2).

**Table 2:** Model QAICc values and weights.

	Model	QAICc	Δ QAICc	Wi	D^2^
Total activity					
	Water	46.45	0	0.21	0.61
	Pk Size	47.21	0.76	0.14	0.55
	Edge	47.96	1.51	0.10	0.49
*Tadarida brasiliensis* activity					
	Edge	47.64	0	0.18	0.63
	Pk Size	47.81	0.17	0.17	0.62
	Water	48.18	0.54	0.14	0.60
*Myotis yumanensis* activity					
	(Intercept)	33.93	0	0.21	
	Native	34.27	0.34	0.18	0.37
	Water	34.95	1.01	0.13	0.29
Species Richness					
	Water	33.29	0	0.26	
	Edge + Lg Pk	34.95	1.66	0.11	

QAICc values, weights, and D^2^ values for the top-ranking models explaining the influence of habitat variables on bat activity for all species, *T*. *brasiliensis* only, and *M*. *yumanensis* only and species richness for 22 parks in San Francisco, California, in 2008–2009. Model rankings were based on Akaike’s Information Criterion corrected for small sample size and overdispersion (QAICc). ΔQAICc is the difference in value between QAICc of the current model versus the best-approximating model (QAIC_min_) for each set of models. W_i_, Akaike weight, is the probability that the current model (i) is the best approximating among those considered for each group [80]. The full set of model results is available in S3 Table.

**Table 3:** Relative importance (and sign of relationship) of model variables for different measures of activity and species richness.

	Total Activity	*T*. *brasiliensis* activity	*M*. *yumanensis* activity	Species Richness
Distance to water	0.49 (-)	0.38 (-)	0.26 (-)	0.66
Park Size	0.33	0.35	0.21	0.26
Forest Edge	0.26	0.38	0.16	0.36
Distance to Lg Park	0.18 (-)	0.19 (-)	0.19	0.36
Pct Native Vegetation	0.19 (-)	0.16 (-)	0.35 (-)	0.21

Relative importance of predictor variables across all models explaining influence of habitat variables on bat activity for all species, *T*. *brasiliensis* only, and *M*. *yumanensis* only, and species richness. Values are the sum of Akaike weights across all 11 models where each variable occurs. Relationships were positive unless designated (-).

**Fig 2:**
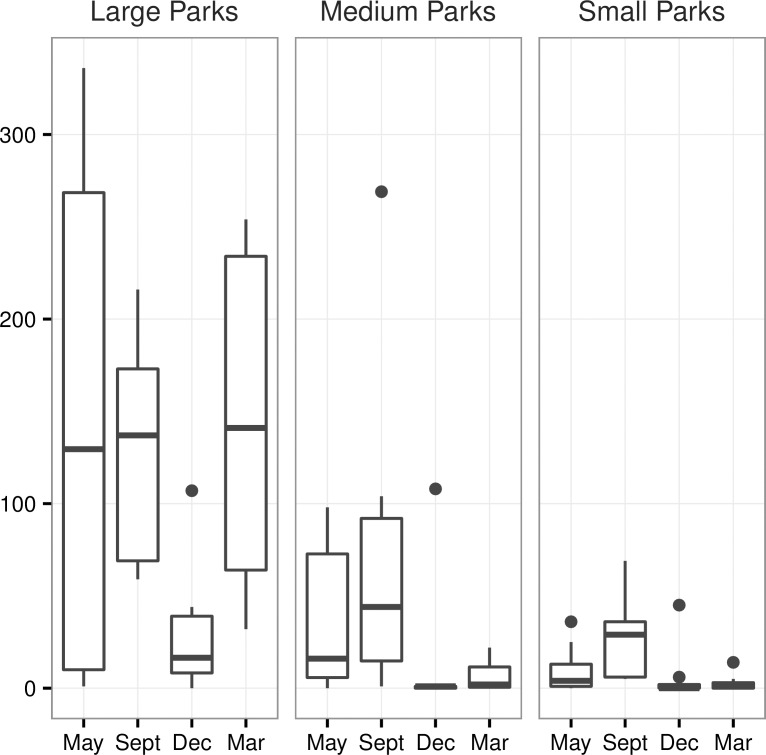
Seasonal activity by park size for all species combined. Total number of bat passes recorded by season. Parks are grouped by size, where large parks > 1,000 ha, medium parks > 100 ha, and small parks < 100 ha.

We detected all four species in only two of the 22 parks, Pine Lake and the Twin Peaks reservoir (S2 Table). No parks had three species. Seven parks had two species (*T*. *brasiliensis* and *M*. *yumanensis* or *T*. *brasiliensis* and *L*. *blossevillii*), and 13 parks had only one species (*T*. *brasiliensis*). Model averaging results show that distance to water was the most important factor explaining species richness (Table 3). Overall activity was highest in May and September, and decreased significantly in December (Fig 2, Tukey mean difference _Dec-Sept_ = -1.54 ± 0.47, Pr(>|z|) = 0.006).

## Discussion

Our results supported our predictions of decreased species richness and are consistent with findings from other studies of urban bat activity. Bat species richness was substantially lower than in the less intensively urbanized surrounding region, with 4 species in San Francisco versus 15 species in the regional pool (Table 1), and was dominated by one species, *Tadarida brasiliensis*. We found that amount of forest edge and proximity to water, factors known to be related to insect abundance [84–86], are important characteristics along with park size in explaining the distribution of bat activity and species richness in San Francisco parks. Finally, we found bat activity year-round, including the presence of transitory migrant species.

### Core urban effects

This study provides insights on the effects of dense urban centers on bats. First, bats persisting in more dense urban areas often have “urban adaptor” traits that enable bats to forage above the canopy or in open areas in natural habitats, such as high wing loading (proportion of bat weight to wing area) and lower frequency echolocation [12, 87], both of which are found in the dominant species in this study, *T*. *brasiliensis*. In cities, these traits allow bats to travel longer distances between foraging sites and to avoid noise, lights, and other urban disturbances [26, 88]. Bats with urban adaptor traits were dominant in this study and in other urban bat studies (but see [23]). Second, urban bat communities appear to differ from taxa subject to biological homogenization such as birds [19], where urban communities often include non-native species, and are independent of regional diversity and more similar to distant urban communities than adjacent areas [89]. In bats, the dominant species differs between urban areas and depends on the regional species pool. For example, *T*. *brasiliensis* was similarly dominant in cities within its geographic range such as Mexico City [22] and Waco [90], but not in cities outside of its range such as Minneapolis [91] or Washington, D.C. [92]. Finally, habitat characteristics describing bat activity in other core urban area studies differed from other taxa as it did in this study [8, 27, 91]. Habitat preferences differed, and sometimes conflicted, between bats and other taxa in the study area. For example, Pine Lake Park in San Francisco is a medium-sized park (37.19 ha) with a small lake and abundant non-native forest. Despite heavy recreational use, it supported the highest bat species richness in the city. This contrasts sharply with results from studies of invertebrate taxa in the same park that showed very low levels of activity and diversity for ants and bees [56, 57].

This study also showed that San Francisco might differ from other core urban areas. For example, there is more extreme reduction in species richness (25% lower) from the species richness in surrounding areas, perhaps due to geographic isolation. In Minneapolis, 86% (at least 6 of the 7) of the bats known in the state occurred in the core urban area [91]. At 25%, San Francisco is more similar to Singapore, which while covering a considerably larger area than San Francisco, only hosts 30%, or 15 of the 50 insectivorous species found on the adjacent peninsula [93]. The results of this study may reflect the combination of geographic isolation and extreme urban density in the study area, and thus provide a glimpse of possible effects of continued increases in urban density worldwide.

### Species richness and dominance

As we predicted, species richness of bats was lower in San Francisco (4 species) than recently documented in San Mateo County, further south on the San Francisco peninsula (up to 13 species, Table 1). There are many possible explanations for this difference. For example, some bats are known to avoid lights [88] and these bats might not tolerate the brightly lit urban environment [94]. Gleaning bats such as *Antrozous pallidus* may be unable to detect their prey over ambient noise in the city [95]. Some bat species avoid busy roadways [96] but in San Francisco bats must fly above busy streets or stay within parks that offer both roosting and foraging habitat. Finally, much of the forested areas in San Francisco consist of eucalyptus or pine species, and lower than expected bat diversity has been documented in forests primarily composed of eucalyptus [97] or pine [50], as has reduced arthropod abundance [98] and bird diversity [99]. Bat species known to forage in mature native forest such as *M*. *evotis* [100], *M*. *thysanodes* [101], and *M*. *volans* [102] may avoid these forests and developed areas. Surveys using different acoustic technology have been unable to distinguish between calls of *M*. *yumanensis* and those of *M*. *californicus*, which has been previously documented in San Francisco [44, 46]. While we should have been able to distinguish between them, it is possible that such calls were overlooked in this study. Finally, it is also possible that additional survey periods or multiple sampling locations per park would have revealed the presence of additional species [103], such as those passing through as migrants such as *Lasionycteris noctivagans*, or bats not easily detected using acoustic sampling such as *A*. *pallidus* and *Corynorhinus townsendii*.

The extreme dominance of *Tadarida brasiliensis* in our study area supports our prediction that urban areas are associated with increased abundance of dominant species. An earlier study in San Francisco also noted dominance of *T*. *brasiliensis* [46] but *T*. *brasiliensis* was not dominant at an undeveloped site 40 km to the south [47]. Our findings are also consistent with other studies reporting dominance of one species in urban areas: *M*. *lucifugus* [23], *Eptesicus fuscus* [32, 91, 92, 104–107], *E*. *serotinus* [25, 108], and *Chalinolobus gouldii* [12]. Like other urban-dominant bats, *T*. *brasiliensis* is adapted for foraging above clutter and over larger areas [109]. It was the dominant species in Mexico City [22] and in Waco, Texas [90]. *T*. *brasiliensis* and other bats in the mollosid family have been characterized as “urban exploiters” [87]. The dominance of *T*. *brasiliensis* in this study may also be an artifact of the greater foraging range of this species. San Francisco is smaller than the documented foraging range of *T*. *brasiliensis* (50 km [110]), and a single bat might visit multiple parks during a single night. Thus, while the number of *T*. *brasiliensis* calls was clearly dominant, we cannot draw any conclusions from this study on actual bat abundance in the study area.

The other species of bats found in this study are associated primarily with water and much smaller foraging ranges (*M*. *yumanensis* [111, 112]), are uncommon in the area (*M*. *lucifugus*, Table 1), or are seasonally uncommon as a migrant (*Lasiurus blossevillii* [47]). We were particularly surprised by the apparent absence of *E*. *fuscus*. Many other studies of bats in urban temperate areas such as Detroit, Denver, Atlanta, Minneapolis, Washington, D.C., Montreal, and Warsaw, Poland report *E*. *fuscus* or its European congener *E*. *serotinus* as being present and often very common [25, 32, 91, 92, 104–107, 113]. *E*. *fuscus* is one of the most widely distributed and commonly detected species in California, reported as common in the Santa Cruz mountains approximately 60 km south of the study area [45] as well as to the north and east across the San Francisco bay [114, 115]. A small number of recordings of *E*. *fuscus* were documented previously in the Presidio of San Francisco [46] but *E*. *fuscus* was not recorded at that location during this study, nor during recording sessions conducted outside of the survey dates included in this analysis. It is possible that additional survey effort may have produced *E*. *fuscus* recordings but in that case, the species is present but rare. The *E*. *fuscus* echolocation call repertoire is variable and can overlap with *T*. *brasiliensis*, so it is possible that some less characteristic *E*. *fuscus* calls were attributed to *T*. *brasiliensis*, but the lack of any typical *E*. *fuscus* calls reinforces our interpretation of their absence in the data set.

Other studies have found reduced *E*. *fuscus* activity in areas with a higher degree of urbanization [22, 30, 91, 92, 105], lower levels of insect abundance [11, 22] and higher levels of pollution [116]. It is possible that the extremely high level of urbanization and low insect levels in the core city area restrict these bats to the suburban areas. In Europe, *E*. *serotinus* was found to roost in buildings but forage outside the city [117], and in Indianapolis, *E*. *fuscus* crossed urbanized habitat to reach foraging areas but did not forage in the urbanized areas [30]. Although *E*. *fuscus* is known to forage above the canopy and in open areas [118], Dixon [91] found them to avoid impervious surfaces and open habitat in Minneapolis. Coleman & Barclay [23] speculated that the smaller size of insects in urban areas [119] could result in less attractive prey for *E*. *fuscus*, a larger bat than the dominant *M*. *lucifugus* found in their study. Thus, the unique geography of San Francisco might make the abundance of potential roost sites not worth the trouble of traveling to find preferred foraging. Another possibility is some form of competitive exclusion of *E*. *fuscus* by *T*. *brasiliensis* as has been suggested for the decline of roosting *Nycticeius humeralis* and concurrent increase in abundance of *E*. *fuscus* in Indiana [120].

### Habitat and seasonal factors

Edge habitat is important for many mammals [121] and the relative importance of edge habitats as a factor explaining bat foraging activity in San Francisco agrees with the results of many other urban studies of bats [36, 37, 40, 87, 113]. Edge habitat has been found to contain more flying insects [84, 85], and since bats tend to be opportunistic foragers [112], edge would thus be more attractive for foraging insectivorous bats. Proximity to water was also an important factor explaining foraging activity and species richness in this study. Like other *Myotis* species [37], *M*. *yumanensis* often forages preferentially over and near water [111, 112].

Park size is often an important predictor of richness and/or abundance of many different urban taxa [2, 122–124]. Avila-Flores and Fenton [22] report a significant positive relationship between activity and park size for *T*. *brasiliensis* in Mexico City. Park size was less important than edge or water for *T*.*brasiliensis* in this study. Since *T*. *brasiliensis* forages over large areas and flies well above the canopy [109], it is presumably not limited to individual parks in San Francisco and can choose or move amongst those with greater amounts of forest edge or water. Finally, the percent of park containing native vegetation was important in explaining foraging activity for *M*. *yumanensis*, which had more activity in parks with less native vegetation. Parks with water in San Francisco are often characterized by non-native plantings (Pearson correlation r = 0.55, p = 0.008), particularly eucalyptus or pine forests. Despite evidence that those forests can harbor lower arthropod abundance [98], this study suggests that non-native vegetation can provide suitable foraging habitat for bats.

All four species of bat found in San Francisco during this study were active during the winter of 2008–2009. Activity was highest in May and September, and was significantly lower in December. In contrast to our results, Pierson and Rainey [46] found *T*. *brasiliensis* activity lowest during the summer months in the Presidio of San Francisco and speculated that *T*. *brasiliensis* overwinter in areas like San Francisco, along the coast, before migrating to the warmer central California valley to breed in the summer. Many populations of *T*. *brasiliensis* in North America are migratory [125], although little is known about migratory movements of *T*. *brasiliensis* in California. In San Diego county, *T*. *brasiliensis* was abundant from March through September but largely absent from known roosts by December [126]. The pattern in *T*. *brasiliensis* activity across seasons found in this study suggests that the bats may leave the core urban area during winter, but studies are needed to understand regional movement of this species in California.

Densely populated core urban areas may represent a population sink even for species that are present and currently abundant. For example, in Calgary, *M*. *lucifugus* dominated core urban areas, but were less healthy than those found in outlying areas [14]. The dominant bat species in San Francisco, *T*. *brasiliensis*, provides valuable pest-control ecosystem services in agricultural areas [127], but very little is known about the role of those or any other ecosystem services in highly urbanized areas [5, 128]. Bats can serve as bioindicators of ecosystem health [129–131]. However, urban bats, poorly understood compared to urban plants, arthropods or birds [132, 133], offer a greater challenge for conservation than these better-known species. In this study, proximity to water was an important factor for both foraging and species richness of bats, and preserving fresh water sources in natural areas should be a part of management priorities. Additionally, factors important for explaining patterns of bat foraging and diversity in San Francisco parks differ from and even contradict factors important to other taxa. Thus, effective conservation efforts aimed at maintaining diversity and ecosystem services function in San Francisco and other increasingly urbanized settings will require a nuanced management strategy.

## Supporting Information

S1 Table Detailed information about San Francisco parks included in the study. (XLSX)

S2 Table Bat activity data. (XLSX)

S3 Table Bat activity models. (XLSX)
